# Comparing international postgraduate training and healthcare context with the UK to streamline overseas GP recruitment: four case studies

**DOI:** 10.3399/bjgpopen20X101034

**Published:** 2020-06-10

**Authors:** Emily Fletcher, John Campbell, Emma Pitchforth, Adrian Freeman, Leon Poltawski, Jeffrey Lambert, Kamila Hawthorne

**Affiliations:** 1 Research Fellow, University of Exeter Medical School, Exeter, UK; 2 Professor of General Practice and Primary Care, University of Exeter Medical School, Exeter, UK; 3 Senior Lecturer and Research Fellow in Primary Care, University of Exeter Medical School, Exeter, UK; 4 Professor of Medical Education, University of Exeter Medical School, Exeter, UK; 5 Royal College of General Practitioners, London, UK

**Keywords:** postgraduate education, education, licensure, appraisal and revalidation, research methods, general practitioners

## Abstract

**Background:**

There are ambitious overseas recruitment targets to alleviate current GP shortages in the UK. GP training in European Economic Area (EEA) countries is recognised by the General Medical Council (GMC) as equivalent UK training; non-EEA GPs must obtain a Certificate of Eligibility for General Practice Registration (CEGPR), demonstrating equivalence to UK-trained GPs. The CEGPR may be a barrier to recruiting GPs from non-EEA countries. It is important to facilitate the most streamlined route into UK general practice while maintaining registration standards and patient safety.

**Aim:**

To apply a previously published mapping methodology to four non-EEA countries: South Africa, US, Canada, and New Zealand.

**Design & setting:**

Desk-based research was undertaken. This was supplemented with stakeholder interviews.

**Method:**

The method consisted of: (1) a rapid review of 13 non-EEA countries using a structured mapping framework, and publicly available website content and country-based informant interviews; (2) mapping of five ‘domains’ of comparison between four overseas countries and the UK (healthcare context, training pathway, curriculum, assessment, and continuing professional development (CPD) and revalidation). Mapping of the domains involved desk-based research. A red, amber, or green (RAG) rating was applied to indicate the degree of alignment with the UK.

**Results:**

All four countries were rated ‘green’. Areas of differences that should be considered by regulatory authorities when designing streamlined CEGPR processes for these countries include: healthcare context (South Africa and US), CPD and revalidation (US, Canada, and South Africa), and assessments (New Zealand).

**Conclusion:**

Mapping these four non-EEA countries to the UK provides evidence of utility of the systematic method for comparing GP training between countries, and may support the UK’s ambitions to recruit more GPs to alleviate UK GP workforce pressures.

## How this fits in

In the context of GP workforce challenges and efforts to recruit doctors from overseas, to the authors’ knowledge, this is the first study demonstrating the application of a systematic methodology for comparing international postgraduate GP training and healthcare context with the UK. Four countries outside of the EEA were systematically compared with the UK using an approach developed to stand up to academic scrutiny. The research provides information to guide future decisions and direction about determining the most efficient and least burdensome route for overseas doctors to enter UK general practice, while maintaining patient safety and registration standards.

## Introduction

As in many western healthcare settings, the UK is currently experiencing a shortage of GPs.^1^ NHS England has committed to recruit an additional 6000 GPs by the end of 2020.^2–5^ As part of the plan to achieve this, NHS England’s International GP Recruitment Programme aims to recruit 2000 GPs from overseas.^3^ This programme also aims to attract UK GPs currently working overseas to return to UK practice.

Under European law, training of GPs from countries within the EEA is recognised as equivalent to that provided in the UK. Such GPs within the EEA applying to practise in the UK are, therefore, given automatic entry to the GP register of the UK’s GMC.^6^ At the time of writing, the proposed departure of the UK from the European Union has the potential to affect this arrangement. Doctors from non-EEA countries apply to join the UK GP register by obtaining a CEGPR.^7^ The CEGPR application process involves an initial assessment by the GMC, the GP providing substantial documentary evidence of their training, qualifications, and experience. This portfolio of evidence is assessed by the Royal College of General Practitioners (RCGP) before a final decision on eligibility is made by the GMC.^8^

The duration and burden of registration processes may be an obstacle to recruitment of GPs from non-EEA countries. Therefore, in order to simplify and streamline these processes, the research team worked in collaboration with the RCGP to develop a systematic methodology for comparing GP training and experience in overseas countries, to determine those that might be broadly equivalent to the UK GP programme.^9^ That initial research included piloting the methodology using the test-case of Australia. This present report details the application of the methodology to four additional countries: South Africa, Canada, the US, and New Zealand. In each of these countries, a structured postgraduate programme exists for the training of GPs or family physicians: a 4-year programme in South Africa; a 2-year programme in Canada; a 3-year programme in the US; and a 3-year programme in New Zealand.

## Method

The research team undertook: (1) a rapid review of 13 non-EEA countries, guided by NHS England, to inform the selection of those for detailed mapping; and (2) application of the mapping methodology to four selected countries: South Africa, Canada, the US, and New Zealand.

### ​Stage 1: Rapid review

A structured framework of questions was applied to 13 non-EEA countries. This involved a rapid collation (over 2 days per country) of publicly available information, including website content, particularly GP training curriculum documentation. The framework questions aimed to establish, for each country, the feasibility and utility of applying the systematic mapping methodology to describe the broader healthcare system in the country of interest and to compare GP training with that of the UK.

The screening framework included questions concerning: (1) the existence of information about the healthcare context and GP training (for example, is there a recognisable model of primary care or general practice or family medicine? Is there an equivalent GP role to that in the UK? Is there a professional membership body? Is information available regarding training and examination?); (2) practical information (for example, are information and documents published in English? Are training curricula available online? Are there multiple curricula?); and (3) current GP workforce context (for example, what is the likely size of the GP pool? Are there workforce shortages?). A team judgement was made regarding whether sufficient information was available to compare the five domains of interest in order to undertake the full mapping exercise described in Stage 2.

### ​Stage 2: Application of the mapping methodology

Four of the countries were shortlisted from the rapid review work as being feasible and potentially useful to map to the UK. NHS England agreed that the following countries should be mapped: South Africa, Canada, the US, and New Zealand.

The research team investigated each country in turn (approximately 6 weeks per country), applying the previously published methods piloted on Australia.^9^ The mapping framework comprises five main ‘domains’ of comparison between the overseas country and the UK as follows:

- domain 1: healthcare context;
- domain 2: GP training pathway;
- domain 3: GP training curriculum;
- domain 4: GP training assessment; and,
- domain 5: CPD and revalidation.

The specific areas within each domain had been previously decided on through stakeholder consultation with the RCGP, GMC, and NHS England.^9^ The mapping of the domains involved collecting data through desk-based research, supplemented by interviews and email correspondence with country-based informants. The research team sought to populate a detailed framework of questions relating to each domain and to provide a commentary on the similarities and differences between the UK and the case-study country of interest. Country-based informants in each country were identified through the RCGP’s professional networks, and also through contacting those listed in lead roles on professional organisation, or GP college websites and/or those who had published relevant work on medical education in each country.

**Figure 1.**
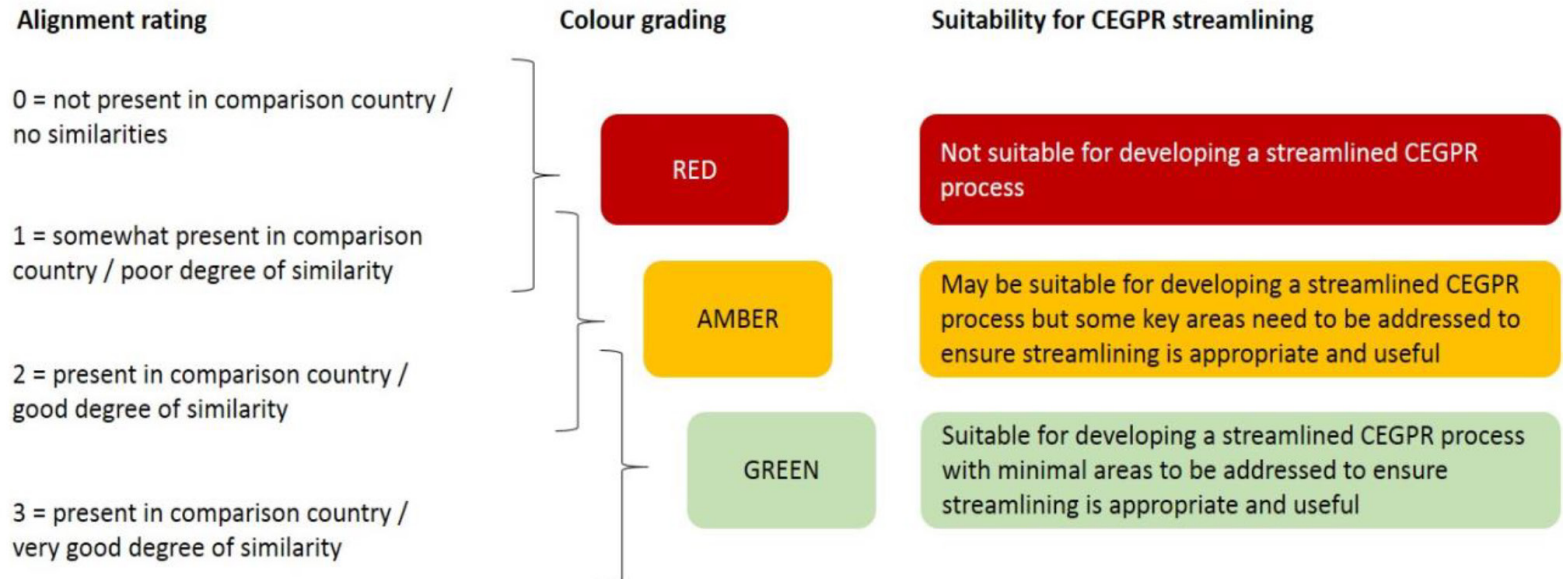
Alignment-rating system (colour). CEGPR = Certificate of Eligibility for General Practice Registration

The final step was to apply an ‘alignment rating’ to each mapping domain (Figure 1) and to generate an overall country rating summarising that country’s alignment to the UK’s GP training programme and the broader healthcare context. The rating involved a RAG rating for each domain, and, in addition, a summarising RAG rating at the level of the country training programme; where a country had two training programmes, these were separately rated. Final alignment and RAG ratings were agreed among the research team, which comprised of both clinical (GP) and non-clinical academic staff, including a GP Professor of Medical Education.

The RAG ratings were intended to act as an indicator of low or moderate or good alignment between systems and training of the country in question with those of the UK. The ratings were not intended to indicate quality of GP training. The overall RAG rating for a particular country related to whether graduates of that country’s GP training programme might be suitable for a streamlined process when considering their eligibility to work in the UK. The rating did not identify the suitability of graduates to undertake work in the UK.

## Results

**Figure 2.**
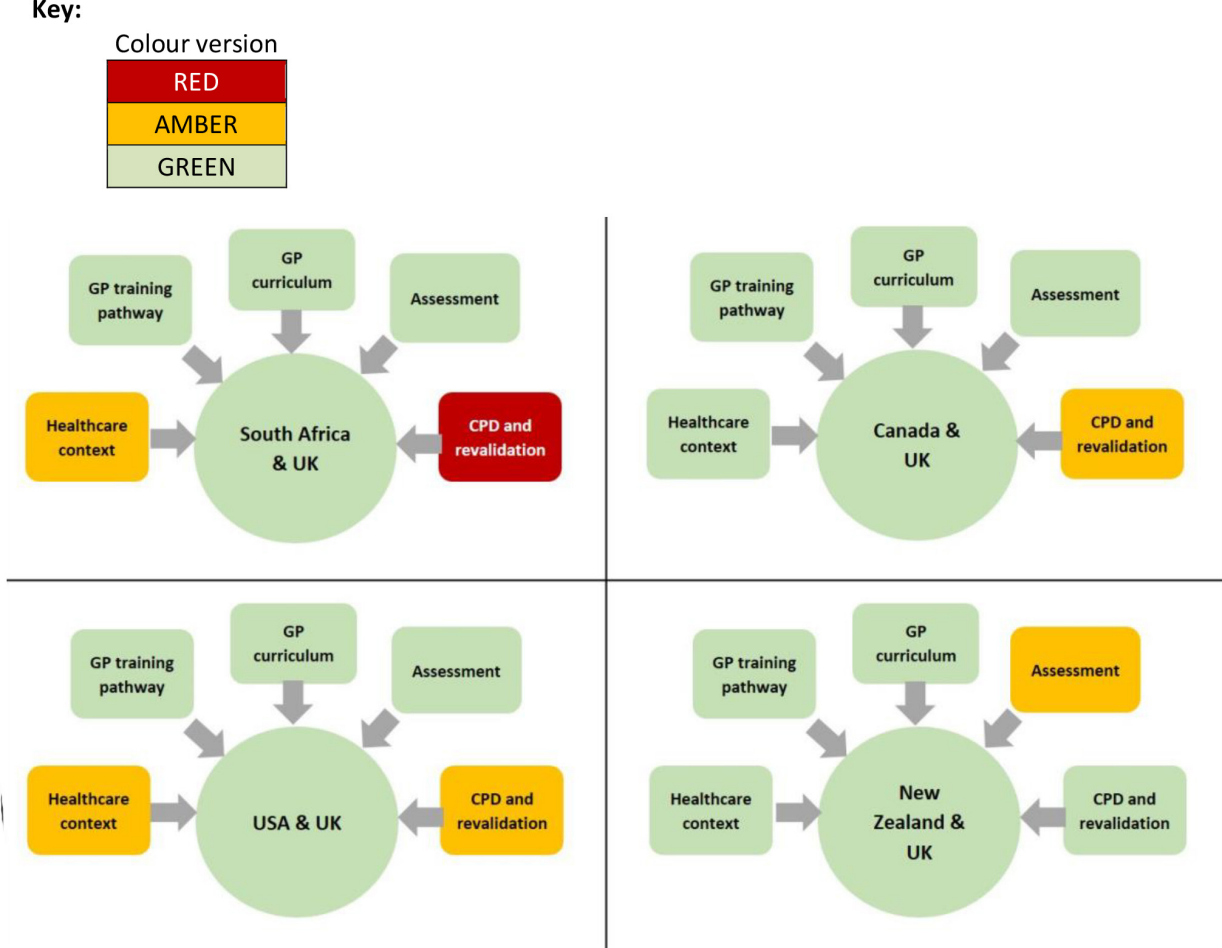
Results of mapping South Africa, Canada, the US, and New Zealand to the UK. CPD = continuing professional development.

Findings from the detailed mapping work showed all four shortlisted countries rated as having ‘good’ overall alignment with the UK. This indicates that there was sufficient evidence that the training and professional experience of graduates from the GP training programmes (or their differently named equivalents) in these countries was broadly equivalent to that obtained in the UK (Figure 2).

However, mapping the five individual domains highlighted some areas of difference to the UK. For example, both South Africa and the US were judged to have low alignment when considering the healthcare context, such as the structure and funding of the healthcare system, and the organisation and role of general practice or family medicine (more information available from the authors on request). Alignment with UK systems in respect of processes relating to CPD and revalidation was also judged to be low for the US, Canada, and, most notably, for South Africa (rated red) where no system of revalidation or recertification currently exists. In addition, assessments undertaken by GP registrars in New Zealand within their training programme showed only moderate alignment with the UK RCGP assessments and examinations. None of the domains showing low or moderate alignment were judged as precluding an accelerated process for admission to UK general practice; for example, it was generally agreed between the research team and RCGP and NHS England that overseas GPs would inevitably need initiation regarding the UK’s NHS and CPD and revalidation processes.

## Discussion

### ​Summary

A rapid review of 13 non-EEA countries indicated four where it would be feasible and potentially useful to undertake a detailed comparison with the UK in respect of GP training and healthcare context (South Africa, Canada, the US, and New Zealand). Application of the systematic mapping methodology to these countries resulted in all being rated as having ‘good’ overall alignment with the UK, despite some individual domains of mapping showing ‘moderate’ or ‘low alignment’.

The overall ratings of ‘good alignment’ appear to offer realistic potential for the introduction of a streamlined evaluation of graduates and those training programmes under the UK’s CEGPR process to ease the route to UK general practice for GPs or family physicians from these countries. A key benefit of the mapping methodology is that it provides detailed insight into the areas of difference between two countries, findings which the RCGP and the GMC may consider when developing and introducing a streamlined CEGPR process for any given country. Such a process has already been introduced for Australia following the authors' pilot work,^9,10^ and similar streamlined processes are now in development for all four of the countries reported in the present work.

### ​Strengths and limitations

The project was undertaken by an experienced academic team providing the project with clinical, primary care, educational, international healthcare, and methodological expertise. A robust methodology was used for mapping the four countries, broadening the applicability of the method that was previously piloted using Australia as a test-case.^9^ The mapping was aided by the availability of the majority of the country-specific information online and presented in English. In addition, communication and interviews with country-based informants based in general practice or family medicine in each country was also conducted in English. To the authors' knowledge, no prior work has involved systematic comparison of GP training between different countries and the UK. The necessity for a rapid turnaround of this work prevented there being time for an in-depth review of each country’s mapping by a country expert, although country-based informants provided valuable input to each report. Integrating an expert review could enhance the value of this mapping approach in its future application. Finally, data extraction and evaluation was undertaken by up to three members of the research team for each country, prior to synthesis by the study manager and consideration by the whole team before reporting; time did not permit dual, independent assessment of data sources. For pragmatic reasons, the authors feel this method is sufficient to proceed with a decision regarding streamlining the CEGPR process, but if this was to be done as an academic research exercise a more rigorous approach would be needed.

### ​Comparison with existing literature

Although published work exists relating to comparison of primary school education curricula in England to those of other countries,^11^ to the authors' knowledge, no prior work has explored the potential for mapping GP training between different countries and the UK.

### ​Implications for research and practice

An ongoing challenge for future application of this methodology is accommodating continuously evolving systems within the mapping domains, such as updates to GP training curricula or changes to regulations concerning CPD and revalidation or recertification. Caution is also needed with interpretation of similarities and differences, even when mapping countries that have documentation in English, owing to differing terminology and concepts across cultures. Mapping of countries that do not use or publish information in English would be a greater challenge owing to the time and cost of translation. Furthermore, data sources do not always use identical definitions of demographic and other variables that were compared between countries.

Broader application of this work is also possible. The methodology provides reassurance that GP training in other countries can be systematically compared with that of the UK, providing there is a structured training programme for GPs or family physicians. It may also be useful in informing any similar efforts by other medical specialties or healthcare professions looking to recruit internationally. It may also be of use in the context of recent work by WONCA Europe and its teaching organisation (European Academy of Teachers in General Practice/Family Medicine [EURACT]) in defining the general practice or family medicine specialty within the European Union.^12^ This approach could also be used by authorities in other countries wishing to streamline their approval processes for foreign applicants. However, it should be noted that the assessment of alignment in this present work is in one direction only. For example, GPs from Canada might meet UK requirements, but perhaps not vice versa and a ‘reverse’ analysis would likely be needed.

The mapping methodology highlights similarities and differences in GP training and the healthcare system context, which could be of importance should GPs or family physicians from these countries wish to enter UK general practice. However, the key statutory, regulatory, and professional agencies involved in GP recruitment and registration must consider the appropriateness of focusing overseas recruitment in countries that are currently facing their own GP or family physician workforce challenges.

Recruiting overseas GPs to support efforts to bolster the UK’s GP workforce will include targeted recruitment from countries outside the EEA, where processes for entry to UK general practice are more complex. The authors' previous work developed a methodology for mapping overseas GP training and healthcare system contextual data to the UK in order to identify the best and most effective and efficient route into UK general practice for those doctors, while seeking to maintain standards and ensure patient safety in service delivery. The methodology has been successfully applied to other countries and produced findings that can be used by individual GPs wishing to move between countries, healthcare providers, and the key statutory, regulatory, and professional agencies responsible for UK medical training and registration. The findings can inform the nature and content of streamlined CEGPR processes for these countries.
